# Green enhancement of wood plastic composite based on agriculture wastes compatibility via fungal enzymes

**DOI:** 10.1038/s41598-022-21705-3

**Published:** 2022-11-10

**Authors:** Mohamed S. Hasanin, Mahmoud E. Abd El-Aziz, Islam El-Nagar, Youssef R. Hassan, Ahmed M. Youssef

**Affiliations:** 1 Cellulose and Paper Department, National Research Centre, 33 El Bohouth St. (Former El Tahrir St.), Dokki, P.O. 12622, Giza, Egypt; 2 Polymers and Pigments Department, National Research Centre, 33 El Bohouth St. (Former El Tahrir St.), Dokki, P.O. 12622, Giza, Egypt; 3 Packaging Materials Department, National Research Centre, 33 El Bohouth St. (Former El Tahrir St.), Dokki, P.O. 12622, Giza, Egypt

**Keywords:** Biotechnology, Materials science

## Abstract

This study deals with the production of natural fiber plastic composites (NFPCs) to reduce environmental pollution with agricultural and plastic waste. Where the NFPCs were prepared from waste/pure polyethylene (WPE) (pure polyethylene (50%)/recycled polyethylene (50%)) and modified sunflower waste via an eco-friendly and economic biological process. The sunflower fibers (SF) were treated via whole selective fungal isolate, namely, *Rhizopus oryzae* (acc no. OM912662) using two different incubation conditions; submerged (Sub), and solid-state fermentation (SSF) to enhance the fibers' compatibility with WPE. The treated and untreated fibers were added to WPE with various concentrations (10 and 20 wt%). The morphology and structure of fibers were characterised by a scanning electron microscope (SEM) and attenuated total reflection-Fourier transform infrared (ATR-FTIR). Furthermore, the mechanical properties, morphology, biodegradation and water vapour transmission rate (WVTR) for the prepared NFPCs were investigated. The results showed that compatibility, mechanical properties and biodegradation of NFPCs were improved by the addition of sunflower fibers treated by SSF conditions.

## Introduction

In the past few years, timber has been the main material for many industries such as the manufacturing of paper, furniture, and packaging boxes, but the increasing need for timbers has become a major contributor to the evanescence of forests^1^. It is known that forests play important role in contributing to climate change mitigation, where they consume approximately 2.6 billion tons of carbon dioxide (CO_2_), which is one-third of the CO_2_ released from burning fossil fuels^2^. Also, agricultural waste is considered a cumbersome by-product, where its disposal by landfilling or incineration is seen as a non-valid solution to the environment^3^. However, agricultural residues are rich in cellulose materials that can be used in various applications as an alternative to forest wood^4–6^.

Moreover, the accumulation of plastic waste (e.g. plastic bottles and bags) in the environment adversely affects wildlife and aquatic life as well as humans. It is known that plastics are cheap and durable, which makes them suitable for different uses^7^, but, due to their chemical structure being resistant to many natural processes of degradation, they are non-degradable^8^.

Wood-plastic composites (WPCs) are considered a subset of a huge class of matter called natural fiber plastic composites (NFPCs). Wood-plastic composites are produced from the fiber or the flour of wood and thermoplastic, while NFPCs are composites of thermoplastic polymers that contain pulp fibers, straw, peanut hulls, coffee husk, and bamboo as a filler^9–11^. The former could be manufactured from agriculture and plastic waste, which is considered an environmentally friendly approach to using agricultural waste and recycled plastic material. Adding cellulosic materials obtained from agricultural waste to plastic materials to prepare WPC enhances their mechanical properties, durability and biodegradability^12^. In addition, WPCs have beneficial characteristics such as low density and cost, durability, high strength, as well as excellent sound-absorbing capacity. So it could be utilized in various applications such as railway coaches, packaging and building^13–15^. Unfortunately, the poor compatibility between fibers (hydrophilic) and polymer (hydrophobic) plays an important role in the properties of produced WPC^16^. Indeed, the compatibility between cellulose and hydrophilic plastics is the main problem facing researchers^17,18^. They, therefore, tend to apply various strategies to overcome this obstacle, which include specific modifications for cellulosic fiber surfaces to minimize their hydrophilicity either by physical or chemical modifications^19,20^.

The volume of sunflower production is about 11 million tons, and the volume of waste generated from its cultivation is about one million tons^21^. Sunflower seeds are used in the production of oil, while the waste is used in many industries, such as livestock feed, compost (alternative soil), water treatment, biodiesel, etc.^22–24^. Sunflower waste was riched by lignocellulosic fibers with high content of lignin about 25%^25^, and so it is difficult to be degradable^25^. Some microorganisms are cabaple to production of enzymes that is able to attack the lignin^26^. Mateusz et al. showed that the addition of sunflower husk to epoxy had a huge effect on the deterioration of flexural strength and tensile of the prepared composite^27^.

Physical treatment of natural fibers includes grinding, thermal and radiation. Grinding is usually used to cut fibers into very small and homogeneous lengths. The thermal treatment is used to change the fibers chemical composition as well as extract of the active components of fibers as a dual-role treatment method. While the radiation treatment includes ultrasonic, plasma, and irradiation treatments^28^. The chemical treatment removes non-desirable components or adds a functional group to improve the compatibility between the fibers and polymer by decreasing the hydrophilicity of the fibers^29^. The chemical modification can also be carried out using benzene diazonium salt, sodium hydroxide and dodecane bromide, or esterification using different fatty acids^30,31^.

In addition, the biological treatment could be used as a tool to reactivate the lignocellulosic fibers surface via purified or crude enzymes as well as other in-suite fermentation conditions using microbial costive agents directly on the substrate with Sub^32^ or SSF^33^. The in-suite treatment offers an economic and eco-friendly process in comparison with other tools with aspects of biological treatment.

In the current work, Sub and SSF conditions were used to activate the surface of the sunflower fibers and modified to become suitable for contact and compactable with a hydrophobic plastic surface. The prepared natural fiber plastic composites (NFPCs) could be suitable for many applications such as alternative wood, household equipment, and packaging.

## Materials and methods

### Materials

All chemicals (Sodium nitrate, dipotassium phosphate, magnesium sulfate, potassium chloride, cetyltrimethylammonium bromide (CTAB) and ferrous sulfate) and media (potato dextrose agar (PDA) medium, potato dextrose broth (PDB) medium and mineral salts medium (MSM)), as well as reagents (3,5-dinitro salicylic acid, pyrogallol, glucose and xylose), were purchased from Sigma-Aldrich in analytical grade without any purification before useing. Pure low density polyethylene (LDPE) was food grade (density 0.93 g/cm^3^, softening point 87.4 °C, and melt flow index (190 °C, 2.16 kg) 6.0 g/10 min), and was obtained from ExxonMobil Chemical (Kingdom of Saudi Arabia) as pallets with particles size ranged 2–5 mm. The polyethylene waste was obtained as pallets with particles size ranged 4–8 mm from Bekia, Egypt.

### Aggregation of fibers

Sunflowers trimmings, as a source of lignocellulosic fibers, were collected from a farm located in El-Menofia Governorate, Egypt, which has considerable amounts of agricultural waste. They were dried in the oven at 70 °C to attain a constant weight and then ground mechanically and sieved with a 200 mesh sieve. The collection of plant material complies with the guidelines of the Ethics Committee in the National Research Centre.

### Fibers treatment

#### Isolation of fungi

Samples for isolation were compiled from the soil of the sunflower farm in Giza, Egypt. Fungal isolation was performed according to our previous work^34^. The isolated fungal was subjected to the cultivation on the replaced Carbone source Czapic broth medium by the fine powder of sunflowers as the sole Carbone source. The selected isolated fungal (*Rhizopus oryzae*) was observed with the highest biomass growth.

#### Identification and characterization of ligninolytic fungus

The selected ligninolytic fungus was identified according to its morphological characteristics and 18s ribosomal DNA (18S rDNA) sequence according to our previous work^35^. The morphological characteristics were examined using a light microscope (Olympus cx41) after 4 days of growth on PDA medium plates via a light microscope at a magnification of 40×. For molecular identification, fungal mycelium from a 4-day-old culture in PDB medium was harvested using Whatman No. 1 filter paper. The total genomic DNA was extracted using the CTAB protocol^36^. The identification was achieved by comparing the contiguous 18S rDNA sequence with data from the reference and type strains available in public databases GenBank using the BLAST programme (National Center for Biotechnology Information). The obtained sequences were aligned using the Jukes Cantor model and the isolate was registered in GenBank^37^.

#### Treatment of sunflower fibers

The sunflower fibers were treated using isolated fungi according to two different incubation conditions. The Sub and SSF conditions were used with the same condition except for the ratio of nutritional medium and fibers^38^. The fungal isolate was cultivated using modified Czapek broth media. The carbon source of the previous media was changed with the lignocellulosic fibers. The submerged fermentation conditions were carried out using 1:25 fibers to medium. The solid state fermentation ratio is 1:5 fibers to medium^39^. The incubation conditions were used for both types of fermentation as follows; temperature 25 °C, initial pH 5.5, and flask volume (1:5) in static condition for 10 days^40^.

#### Lignocellulolytic enzymes inspections

The liquid filtrate from different incubation times (7 days) old fungal cultures cultivated on PDB medium or liquid MSM amended with waste as the sole carbon source were collected and applied directly as the crude enzyme in enzymes assay experiments. The activity of cellulase and xylanase enzyme was carried out via reducing sugars estimations using 3,5-dinitro salicylic acid (DNS) assay with glucose for cellulase and xylose for xylanase^41,42^. Lignin peroxidase activity was determined spectrophotometrically at 420 nm^43^. All appeared values are the average of triplicate experiments.

### Production of natural fiber plastic composites (NFPCs)

The fibers were dried at 60 °C for 12 h to remove the moisture content. Also, the recycled PE was washed with distilled water and dried at 60 °C overnight.

The NFPCs were synthesized from pure LDPE and recycled PE (WPE; 50:50 wt%) which was loaded with untreated fibers, and treated fiber either by Sub and SSF condition at concentration 10 wt% to obtain NFPC10%SF, NFPC10%Sub, and NFPC10%SSF, respectively, and at concentration 20 wt% to obtain NFPC20%SF, NFPC20%Sub, and NFPC20%SSF, respectively. The **c**omposition of the prepared samples is shown in Table 1. The PE was melted in a twin-screw extruder (Haake RheomexTW100, USA) at 140 °C and a rotation speed of 60 rpm. After the PE was melted, the sunflower fibers were added and mixing was continued for an additional 10 min. The mixture was collected and left for cooling then cut into small pieces suitable for feeding into a stainless-steel pressure mould to make plates with dimensions 8 × 10 cm and 0.5 mm thick. The moulding was done using pressure (5 MPa) at 150 °C and for 5 min. after that; the plates were left to cool in the piston to room temperature under pressure. Three replicates were performed for each blend.

**Table 1:** The **c**omposition of the prepared samples (the value by wt%).

Sample	Low density polyethylene	Recycled polyethylene	Sunflower fibers (SF)	Treated fibers via submerged (Sub)	Treated fibers via solid-state fermentation (SSF)
Blank LDPE	100	–	–	–	
Blank WPE	50	50	–	–	–
NFPC10%SF	45	45	10	–	–
NFPC20%SF	40	40	20	–	–
NFPC10%Sub	45	45	–	10	–
NFPC20%Sub	40	40	–	20	–
NFPC10% SSF	45	45	–	–	10
NFPC20% SSF	40	40	–	–	20

### Characterization

The Attenuated total reflection Fourier-transform infrared spectroscopy (ATR-FTIR) of samples was measured on the Shimadzu 8400S FT-IR Spectrophotometer in the range of 500–4000 cm^−1^. The samples were measured as films. In addition, a scanning electron microscope (SEM; JSM 6360LV, JEOL/Noran) was used to study the surface morphology of the prepared samples.

### Mechanical properties

The mechanical characterization was carried out for three replicates of each prepared NFPCs to gauge the tensile strength, elongation, Young’s modulus of bending, and modulus of rupture according to the ASTM D638-91 utilizing a testing machine LK10k (Hants, UK) fitted with a 1kN load cell and operated at a rate of 5 mm/min.

### Biodegradation of NFPCs

Biodegradation of NFPCs in soil was done a corroding to Dalev et al.^44^. The soil was taken from the surface layer, and then all inert materials were removed to obtain a homogeneous mass. About 100 g of soil was poured into a plastic pot up to a thickness of about 3 cm. The prepared samples were accurately weighed and dried for 24 h at 50 °C, then were buried in the pots to a depth of 1 cm. Water was sprayed once a day to sustain the moisture. The samples were weighed weekly for 4 weeks after being washed with distilled water and dried at 50 °C for 24 h. The biodegradation was carried out for three replicates for each sample.

### Water vapor transmission rate (WVTR)

WVTR was carried out using (GBI W303 (B), Water Vapor Permeability Analyzer; China) via the cup method for three replicates of each prepared NFPCs. The WVTR was designed by way of the quantities of water vapor transferred through a unit area of the NFPCs film in a unit time inferior to particular conditions of temperature (38 °C) and humidity (4%) as specified through the following standards (ASTM E96).

## Results

### Identification of isolated fungal

**Figure 1:**
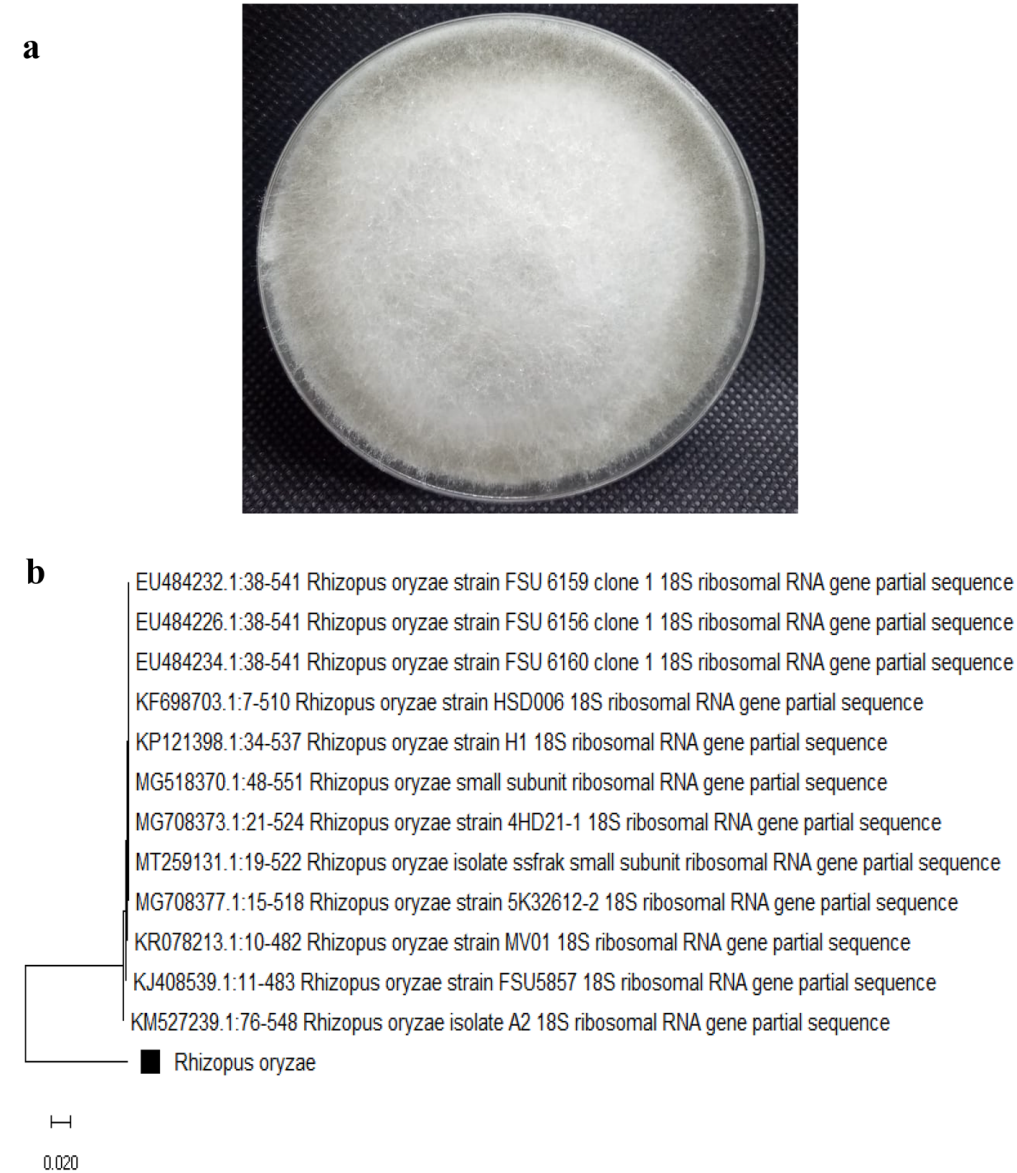
Plate of the pure isolate growth morphology (**a**) as well as the identification tree of molecular identification (**b**). *Rhizopus oryzae.*

The isolated fungal was identified via molecular techniques and morphological characterization. Figure 1a showed that the colonies of isolated fungal contained on the surface of the PDA medium a yellow-green spore on the upper surface and reddish-gold on the lower surface. The genomic DNA of the isolated fungal was extracted and identified via molecular techniques where 18s rRNA amplification was applied. The obtained sequences were compared with related sequences on the National Centre for Biotechnology Information (NCBI), Egypt database. It was found that it was closely associated with *Rhizopus oryzae* (acc no. OM912662) which confirms their morphological identification. Figure 1b illustrated the phylogenetic tree with a high similarity of about 91.37%.

### Enzymes assay

**Figure 2:**
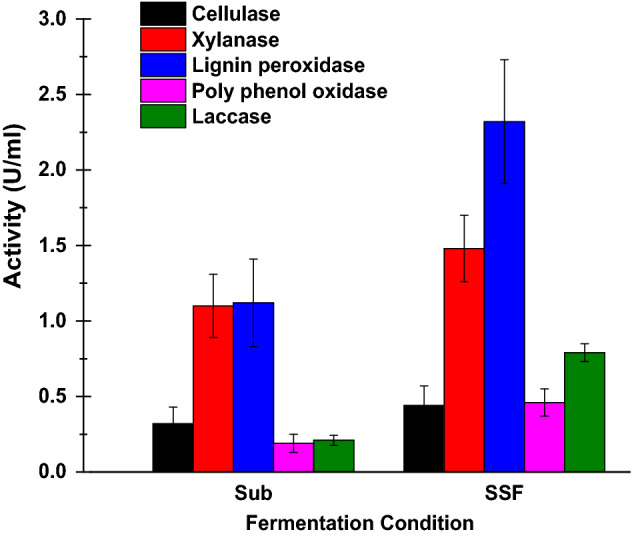
The lignocellulosic enzymes assay for submerged (Sub) and solid stated fermentation (SSF) conditions.

The fungal isolate was subject to two different cultivated conditions due to the ratio between fibers and liquid medium. The quantitative activity of lignocellulolytic enzyme clusters was assayed and shown in Fig. 2. All assayed enzymes in the SSF condition were confirmed as high value in comparison with the other ones produced in the submerged conditions. These results are referred to the harsh conditions which made the isolated fungi produce all enzymes possible to provide the carbon requirements needed to grow. On contrary, in the submerged condition, the fungal isolate grows in normal conditions with a high water content which has many soluble simple carbons dissolved from the fibers^34^. These phenomena induced heavy enzyme production in solid-state fermentation conditions as well as feedback on the production of the enzymes in case of submerged incubation conditions. Moreover, the high productivity of the lignocellulolytic enzymes lead to fiber surface modifications and made it more compatible to interact with polymer plastic where these enzymes increase the hydrophobicity of the fibers. The lignin oxidative enzymes, including lignin peroxidase, polyphenol oxidase and laccase act as natural surface activators where the oxidative effect of these enzymes act as a surface modification process as well as increase surface compatibility to plastic polymer. Whereas, the enzymes eliminated the fine terminal of the fiber molecular structure which made fibers smoother as well as reduced the free active hydroxyl groups and so more hydrophobic^45^.

### Fibers characterization

**Figure 3:**
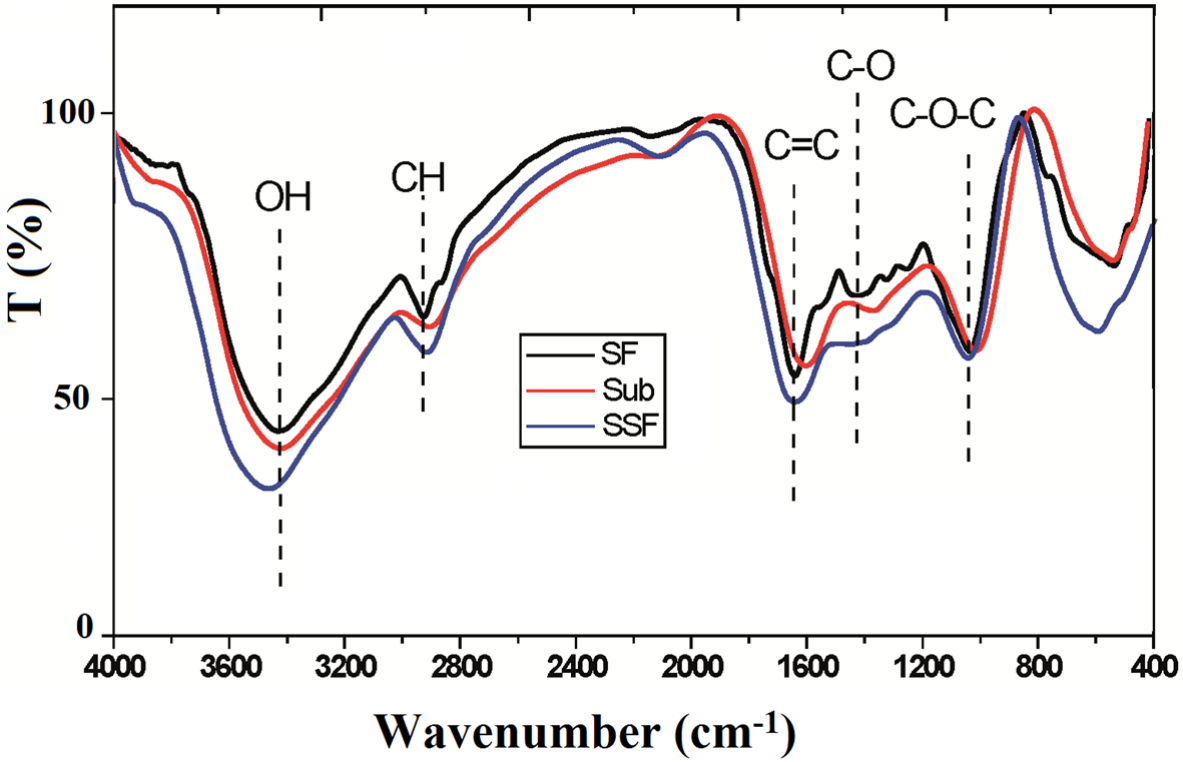
ATR-FTIR of untreated and treated sunflower fibers.

The ATR-FTIR spectra of sunflower fibers and treated ones were shown in Fig. 3. In general, all the untreated and treated fibers showed the broad band corresponding to hydroxyl group (–OH) that stretched at 3400 cm^−1^, C–H stretched at 2900 cm^−1^, C–O–C stretched at 1125 cm^−1^ and C=C stretched alkene at 1610 cm^−1^^38,46^. While the band of C–OH that stretched at 1435 cm^−1^ was present in untreated fibers and disappeared completely in SSF. The disappearance of these bands could have been caused by the partial removal of hemicellulose and lignin from fibers during the fungal treatment^47^. The fungal treatment reduces the hydroxyl groups and so high water resistance through a reaction with enzymes. This led to a decrease in hydrogen bond that increased the intensity of the peak between 3300 and 3500 cm^−1^ bands in treated fibers compared to untreated fibers. Werchefani et al. observed that fibers treated by enzymes have fibers lower in diameter and length as well as high water resistance as a result of elimination of the hydrophilic components (lignin, pectin and hemicellulose)^48^.

**Figure 4:**
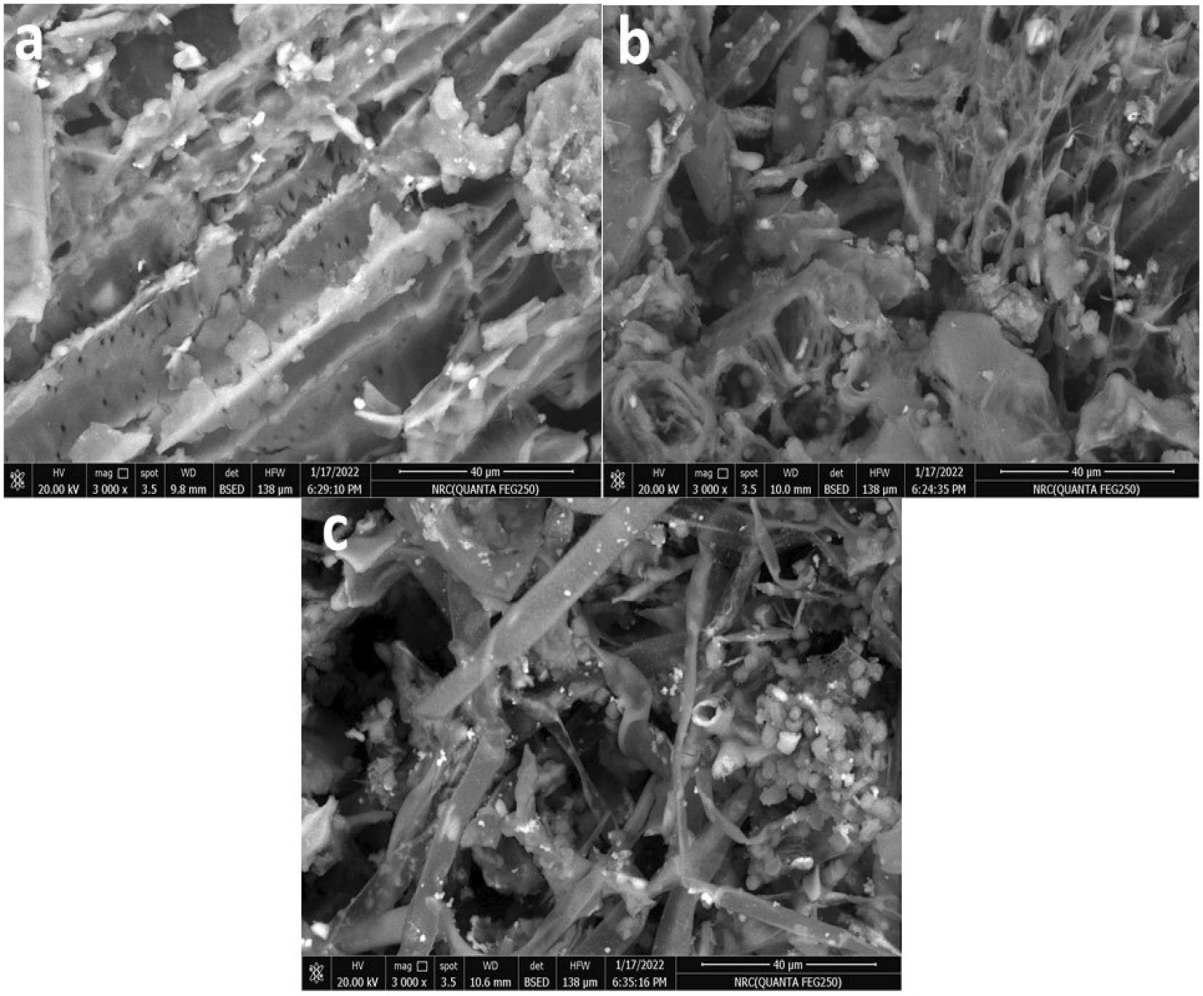
SEM image of blank sunflower fibers (**a**), and the treated sunflower fibers either via Sub conditions (**b**) or via SSF conditions (**c**).

The sunflower fibers consist of many components each of them having a unique performance in SEM imaging. Figure 4 illustrated the effect of the fungal different fermentation conditions on the fibers' surface morphology in comparison with the blank one. The blank and treated fibers observed a significant difference in the surface morphology level which may affect the compatibility of fibers and plastic behaviour. The blank fibers (Fig. 4a) are clear with a typical lignocellulosic fibers performance as smooth surface fibers loaded with some impurities observed as aggregated crystals. Otherwise, the SSF fibers surface in Fig. 4b observed with many pours located overall fibers surface as well as no smooth appearance enough in comparison with a blank one.

In addition, the Sub condition fibers are shown as blank fibers with low smoothness, and the diameter of the fibers was clearly reduced. These observations may be related to the effect of SSF conditions in which the productivity of the fungal enzyme is higher than in the Sub condition according to harsh conditions. Moreover, in the SSF condition, the fungal hypha has penetrated the fibers to achieve the nutrients in harsh conditions (Fig. 4c). These results affirmed that the enzyme productivity, as well as fungal hypha biomass, are increased in the harsh conditions in which the fungal strain combat to gain the required nutrients and survive via degradation of the fibers.

In contrast, the high humidity condition in the Sub case made fungal strains grow at a normal rate. Overall, the revised conclusion was concise that the treated fibers may affect the compatibility with plastic polymer and made the fibers attached strongly with plastic polymer in comparison with the blank fibers.

### NFPCs characterization

#### Mechanical properties

Table 2 represents the effect of untreated and treated sunflower fibers content in the prepared wood plastic composites on the tensile, elongation, Young’s modulus of bending, and modulus of rupture. In general, the addition of sunflower fibers to NFPCs decreased the tensile and elongation of the prepared wood plastic composites in comparison with pure NFPCs. However, the tensile and the elongation of NFPCs containing sunflower fibers modified by the SSF condition were higher than that modified by Sub condition or unmodified fibers. This may be due to the high compatibility between modified fibers (SSF) and the polymer, which was higher than modified fibers (Sub) than unmodified sunflower fibers and the polymer.

**Table 2:** Mechanical properties of the NFPC.

Sample	Tensile strength (MPa)	Elongation (mm)	Young's modulus of bending (MPa)	Modulus of rupture (MPa)
Pure LDPE	12.4 ± 1.2	13.4 ± 1.5	594 ± 30	19.7 ± 1.2
WPE	17.3 ± 1.9	13.4 ± 1.2	755 ± 28	17.6 ± 1.3
NFPC10%SF	6.4 ± 0 .8	13.2 ± 1.3	857 ± 26	15 ± 1.1
NFPC20%SF	6.9 ± 0.55	13.1 ± 1.0	898 ± 31	15.3 ± 0.8
NFPC10%Sub	7.7 ± 0.6	13.1 ± 1.1	875 ± 29	15.7 ± 0.7
NFPC20%Sub	8.4 ± 0.48	13.0 ± 0.9	996 ± 32	16.6 ± 0.9
NFPC10%SSF	7.9 ± 0.40	13.3 ± 1.0	989 ± 15	16.5 ± 0.5
NFPC20%SSF	8.8 ± 1.0	13.2 ± 0.8	1032 ± 33	18.8 ± 0.9

It was found that the content of untreated and treated sunflower fibers increases Young’s modulus of bending NFPCs compared to blank PE related to the increase of the elasticity. These results affirmed the elongation results that emphasised that the addition of fibers was lead to decreases in the elasticity where the relation between elongations, Young’s modulus is inverse. In addition, the treated fiber via SSF conditions is more effective on Young’s modulus (1032.1 MPa) of NFPC that contains 20% SSF. Also, the NFPC contains fiber treated by SSF conditions with a content of 20 wt% improves the modulus of rupture to be 18.8 MPa compared to other fibers, which are near to blank PE 19.7 MPa. This may be due to the compatibility between treated sunflower fibers and the polymer higher than that between untreated sunflower fibers and the polymer. In the same way, Sobczak et al.^49^ reported that as the degree of surface roughness increases in the fiber, the area available for interactions with the matrix will be increased. This leads to improving the mechanical performances of the prepared composites.

#### Morphological properties of prepared NFPCs

**Figure 5:**
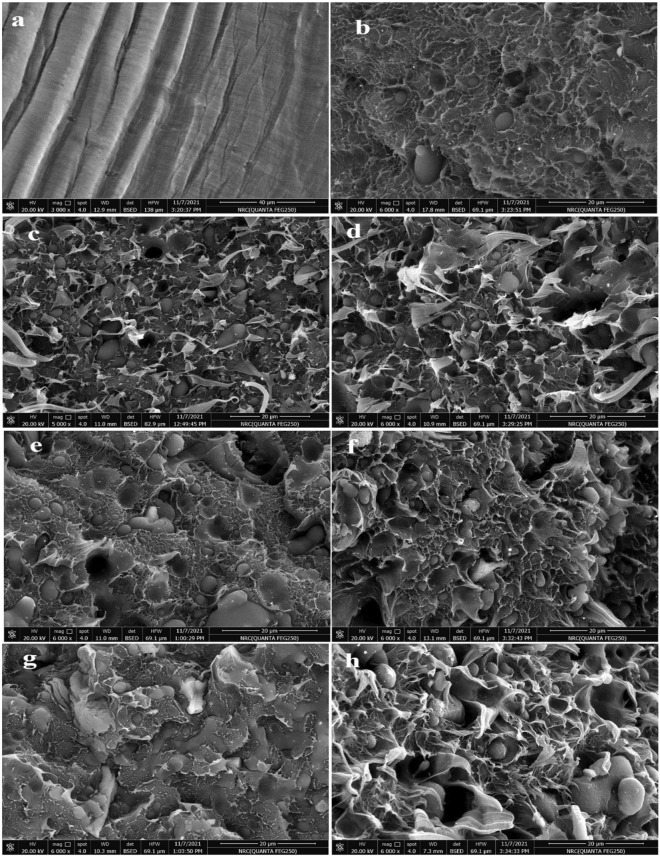
SEM image of pure LDPE (**a**), WPE (**b**), NFPC10%SF (**c**), NFPC20%SF (**d**), NFPC10%Sub (**e**), NFPC20%Sub (**f**), NFPC10%SSF (**g**), and NFPC20%SSF (**h**).

The surface morphology of the prepared NFPCs has illustrated in Fig. 5. The surface morphology of blank PE films was smooth and showed high homogeneity between their polymeric chains (Fig. 5a). This homogeneity was decreased in WPE (50% pure PE/50% recycled PE) as shown in Fig. 5b due to the difference in the polymeric chain's length between pure PE and recycled one, in other words, due to the difference in the molecular weight between pure PE and recycled one^50^. Also, the presence of different additives, impurities, or traces of other polymers in the polymer waste significantly affected on the melt flow index and the homogeneity between virgin and recycled polymer as reported by Patrizio et al.^51^. The loading of WPE by untreated and treated sunflower fibers displayed a rough structure which was decreased in the case of treated fibers, especially with that treated by SSF condition (Fig. 5g). This indicates the happening of compatibility between polymer matrix and treated sunflower fibers. Additionally, the content of fiber in the matrix has a great effect on the surface morphology and so their internal construction.

Indeed, the surface of untreated fibers is enveloped by lignin and some impurities of terminal groups of cellulose and hemicellulose^52^ which is the reason for decreasing the adhesion and so the compatibility between the fibers and WPE (Fig. 5c,d). Whilst, the treated fibers either submerged (Fig. 5e,f) or solid-state fermentation (Fig. 5g,h) showed higher compatibility with polymer. These results may be due to a decrease in the proportion of lignin via oxidization of its phenolic groups via peroxidases enzymes as well as the elimination of terminal side group of cellulose and hemicellulose and their decrease the hydrophilicity via acting the eradication of free hydroxyl groups and these observasions are in a nice agremment with other studies^53,54^, especially those treated by the SSF method as shown in Figs. 3 and 4.

Corradini et al.^55^ showed that the compatibility between recycled poly(ethylene terephthalate) and sugarcane bagasse fiber was increased by the addition of ethylene/n-butyl acrylate/glycidyl methacrylate copolymer as compatibilizing agent that worked to raise the interaction between the components. Also, Chen et al.^56^ illustrated that the alkali treatment of sugarcane bagasse increases their compatibility with high-density polyethylene than the untreated one.

#### Biodegradability

**Figure 6:**
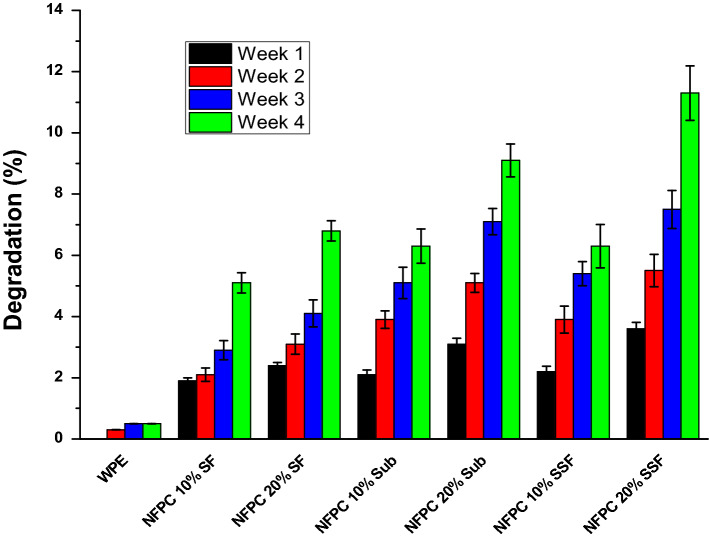
Biodegradability of WPE and the prepared NFPCs.

In the last decay, biodegradability is an important feature for any new component especially the synthetic polymer that has taken a century to start degradation. The addition of natural fibers to synthetic polymers made it more acceptable to degrade in the environment. The biodegradation of the WPE and the prepared NFPCs was illustrated in Fig. 6. The obtained results investigated that the WPE is not degraded in the soil after a complete 4 weeks.

The biodegradability properties of polymers are a key factor to consider this polymer is environmentally friendly and not accumulated over. PE is not a biodegradable polymer that is not biodegraded in the environment naturally while taken to complete degradation many years. Otherwise, natural fibers are biodegradable and environmentally friendly. In this context, cellulose has a degradation half-life (t_1/2_) in the soil at 10–20 °C between 30 and 42 days. Moreover, after 2 months cellulose was decomposed into CO_2_ and water. The fast degradation rate of natural fibers is attributed to the breakdown of cellulose bonds randomly as the effect of the microorganism cleavage^57^. In this work, the effect of the addition of the sunflower fibers on the biodegradability rate of PE in soil was studied and the results are presented in Fig. 6. The obtained results were observed that the sunflower fibers significantly affect the biodegradation of the polymer in soil. Whereas, LDPE offered resistance to biodegradation with lost about 0.18% of its weight after being buried in soil for 4 weeks. Additionally, the sunflower fibers enhanced the composite biodegradability where NFPCs containing 10 and 20 wt% of untreated sunflower fibers (SF) lost about 5.2 and 6.9%, respectively, after the same period buried in the soil. On the other hand, the fungal enzymes treated fibers (Sub) observed a slight increase in biodegradability rate in comparison with blank ones. However, NFPCs containing 10 and 20 wt% of treated sunflower fibers (SSF) lost about 6.4 and 11.4%, respectively, after the same period buried in the soil. These increases in the biodegradability rate of treated fibers composite in soil may be related to the removal of undesirable sunflower fibers constituent that made the fiber easy to microbial attack. These results are in agreement with previous findings on the biodegradability of non-degradable polymer reinforced with sunflower fibers where enhanced tensile strength properties as well as biodegradability^38^. Additionally, the addition of PE did not prevent the biodegradability of sunflower fibers as well as the biodegradation of lignocellulosic material with time which may be made the degradation of PE easy in comparison with the pure one.

#### Water vapor transmission rate (WVTR) of NFPCs

Food degradation is meaningfully affected by the WVTR of packaging materials. It designates together the solubility of water molecules as well as the transfer of water molecules into packaging materials. Hence, the materials' high permeability to water vapor has a straightforward impact on their usage in packaging applications attributable to their capability to alter the block humidity between products and their neighboring air. Several issues contain chemical structure (high solubility for selecting polymer), size, molecular weight, etc. require an influence on moisture content as well as molecule mobility in the packaging films.

Table 3 represents the values of the WVTR of the pure LDPE, WPE, NFPC10%SF, NFPC20%SF, NFPC10%Sub, NFPC20%Sup, NFPC10%SSF, and NFPC20%SSF were shown in (Table 3). The achieved data shows that the WVTR rises in the fabricated NFPCs films with the addition of different ratios of treated fibers (SF, SSF, and Sub fiber) from 10 to 20%. Where the WVTR was increased from 2.73 g/(m^2^ day) for pure LDPE to 4.99 g/(m^2^ day) in the case of using (50:50) percentage of pure LDPE to recycled PE (WPE). Moreover, the WVTR was increased from 4.99 g/(m^2^ day) to 26.37 g/(m^2^ day) in case of using 10% of (SF) fibers. Also, the WVTR increased from 4.99 g/(m^2^ day) to 34.64 g/(m^2^ day) in the case of using 20% of (SF) fibers.

**Table 3:** WVTR of pure LDPE, WPE as well as the prepared NFPCs containing different concentration of unmodified (SF) and modified (Sub and SSF) sunflower fibers.

Sample	WVTR (g/m^2^ day)
Pure LDPE	2.73 ± 0.09
WPE	4.99 ± 0.32
NFPC10%SF	26.37 ± 1.13
NFPC20%SF	34.64 ± 1.34
NFPC10%Sub	16.60 ± 0.69
NFPC20%Sub	29.67 ± 1.28
NFPC10%SSF	12.58 ± 0.57
NFPC20%SSF	15.37 ± 0.50

By using from 10 to 20% of treated fibers (Sub) for the fabrication of the NFPC film, the WVTR increased from 16.60 g/(m^2^ day) to 29.67 g/(m^2^ day). Furthermore, by usage from 10 to 20% of treated fibers (SSF) for fabrication of the NFPC film, the WVTR increased from 12.58 g/(m^2^ day) to 15.37/(m^2^ day). The principal causes for the rise of WVTR with the addition of cellulose fibers are the hydrogen bonds formed between (OH groups) of fibers and the polymer matrix.

The increase in WVTR at higher SF concentration is related to the pore network and structure of the NFPCs films. It was realized that, generally, the WVTR improved with increasing treated fibers (Sub and SSF) ratios in the NFPCs samples. Moreover, the enhancement in the WVTR at higher humidity levels is associated with the improved passage of moisture. This phenomenon is convinced by the transmission of water molecules in the microscopic pores of the fiber material which are filled with water because of capillary condensation^58^. For ingredients that display hysteresis in their sorption isotherm, it has been described previously that their WVTR is dependent on moisture content^59,60^. We detected that there is a small increase in water vapor transmission rate as the percentage of different ratios of treated fibers (Sub and SSF) from 10 to 20% increases. This is as the percentage composition of modified cellulose increases, the hydrophilicity of the NFPCs films increases. This phenomenon could be associated with the significant hydrogen bonding interaction with water.

## Conclusion

The current manuscript established an inexpensive as well as sustainable approach for fabricating wood plastic composites from recycled PE waste as binding matrix and agricultural waste such as modified sunflower waste as filled materials through a biological unusual procedure designated as a green eco-friendly, and economic method. The sunflower fibers were treated via whole selective fungal isolate, *Rhizopus oryzae* (acc no. OM912662), to modify the fibers' surface and to improve their compatibility with polymer plastic by increasing the hydrophobicity of the fibers. The mechanical properties were improved by the addition of both forms of modified sunflower (Sub), and (SSF). Moreover, the untreated and treated sunflower fibers increase Young’s modulus of bending NFPCs compared to blank PE. Furthermore, the treated fiber via SSF conditions is more effective on Young’s modulus (1032.1 MPa) of NFPCs that contains 20% SSF. As well, the fiber treated by SSF conditions with a content of 20%/wt develops the modulus of rupture to be 18.8 MPa compared to other fibers, which are near to blank PE 19.7 MPa. The addition of modified sunflower waste fiber enhanced the composite biodegradability where NFPCs containing 10 and 20 wt% of untreated sunflower fibers (SF) lost about 5.2 and 6.9%, respectively, while the treated fiber by Sub condition showed a slight increase in biodegradability. However, NFPCs containing 10 and 20 wt% of treated sunflower fibers SSF condition lost about 6.4 and 11.4%, respectively, after the same period buried in the soil. Moreover, WVTR increases in the prepared NFPCs films with the addition of different ratios of various fibers from 10 to 20%. Thus, the fabricated wood plastic composites might be appropriate for many applications, e.g. alternative wood, household equipment, as well as packaging.

## Data Availability

The datasets generated during the current study are available in (https://www.ncbi.nlm.nih.gov/search/all/?term=OM912662).
